# Influenza A (H5N1) Viruses from Pigs, Indonesia

**DOI:** 10.3201/eid1610.100508

**Published:** 2010-10

**Authors:** Chairul A. Nidom, Ryo Takano, Shinya Yamada, Yuko Sakai-Tagawa, Syafril Daulay, Didi Aswadi, Takashi Suzuki, Yasuo Suzuki, Kyoko Shinya, Kiyoko Iwatsuki-Horimoto, Yukiko Muramoto, Yoshihiro Kawaoka

**Affiliations:** Author affiliations: Airlangga University, Surabaya, East Java, Indonesia (C.A. Nidom);; University of Tokyo, Tokyo, Japan (R. Takano, S. Yamada, Y. Sakai-Tagawa, K. Iwatsuki-Horimoto, Y. Muramoto, Y. Kawaoka);; Ministry of Agriculture, Jakarta, Indonesia (S. Daulay);; Agriculture and Livestock Agency, Tangerang, Indonesia (D. Aswadi);; University of Shizuoka, Shizuoka City, Japan (T. Suzuki, Y. Suzuki);; Chubu University, Kasugai City, Japan (Y. Suzuki); Kobe University, Kobe, Japan (K. Shinya, Y. Kawaoka);; University of Wisconsin, Madison, Wisconsin, USA (Y. Kawaoka)

**Keywords:** Influenza A virus, H5N1 subtype, Indonesia, pigs, phylogeny, evolution, infection, epidemiology, viruses, research

## Abstract

Pigs have long been considered potential intermediate hosts in which avian influenza viruses can adapt to humans. To determine whether this potential exists for pigs in Indonesia, we conducted surveillance during 2005–2009. We found that 52 pigs in 4 provinces were infected during 2005–2007 but not 2008–2009. Phylogenetic analysis showed that the viruses had been introduced into the pig population in Indonesia on at least 3 occasions. One isolate had acquired the ability to recognize a human-type receptor. No infected pig had influenza-like symptoms, indicating that influenza A (H5N1) viruses can replicate undetected for prolonged periods, facilitating avian virus adaptation to mammalian hosts. Our data suggest that pigs are at risk for infection during outbreaks of influenza virus A (H5N1) and can serve as intermediate hosts in which this avian virus can adapt to mammals.

## Abstract 2

TOC summary: Pigs may serve as intermediate hosts in which this avian virus can adapt to mammals.

A highly pathogenic avian influenza virus A (H5N1) was first recognized among geese in Guangdong Province, southern People’s Republic of China, in 1996 (1). Within a year, this goose virus underwent reassortment with viruses circulating in other avian species. By 1997, the virus had become widespread among poultry in Hong Kong, and direct avian-to-human transmission of influenza A (H5N1) viruses was reported (2,3). Since late 2003, influenza A (H5N1) viruses have spread to domestic poultry in other Southeast Asian countries (4). Since mid 2005, they have been detected across Asia, Europe, and Africa, causing severe damage to the poultry industry and infecting >490 humans, resulting in a mortality rate of 60% (5–8). Indonesia has been particularly affected by these viruses; >160 cases of human infection (i.e., about one third of the total confirmed human influenza A (H5N1) infections worldwide) and a mortality rate >80% have been reported (8). Hence, understanding prevalence and adaptation of influenza A (H5N1) influenza viruses in Indonesia is crucial.

Influenza viruses attach to host cells by binding their hemagglutinin (HA) to cell-surface oligosaccharides containing a terminal sialic acid. The HA of avian influenza viruses preferentially binds to sialic acid linked to galactose by α-2,3 linkages (SAα2,3Gal); that of human viruses binds to SAα2,6Gal (9). Correspondingly, epithelial cells in the upper respiratory tracts of humans mainly bear SAα2,6Gal receptors (10,11), and those in duck intestines (the major replication site for duck viruses) mainly possess SAα2,3Gal (12). Virus receptor specificities and expression patterns of receptors on host cells are thought to be major determinants of the host range restriction of influenza viruses (13). Thus, the recognition of human-type receptors by avian viruses appears to be necessary for these viruses to replicate in the upper respiratory tract and be transmitted efficiently from human to human. Given that influenza A (H5N1) viruses isolated from humans are not transmitted efficiently despite their ability to recognize human-type receptors (14), mutations in the polymerase and other viral genes may also be needed for replication of influenza A (H5N1) viruses in the upper respiratory tract (15).

Traditionally, pigs have been considered as “mixing vessels” (16–19) because they support replication of avian and human influenza viruses (17). Their tracheal epithelial cells reportedly bear SAα2,3Gal and SAα2,6Gal receptors (18). However, recent studies have shown that despite SAα2,3Gal and SAα2,6Gal receptors in pig respiratory tracts, SAα2,3Gal is found only in the smaller airways (bronchioli and alveoli) and not in the trachea (20,21). Kuchipudi et al. (22) found SAα2,3Gal and SAα2,6Gal receptors in the bronchi, bronchioli, and alveoli of chickens and ducks; however, SAα2,6Gal was dominant in chicken tracheal epithelium, and SAα2,3Gal, in duck trachea. Given that influenza A (H5N1) viruses have been transmitted directly from birds to humans, the central dogma of pigs as a mixing vessel may no longer stand. Moreover, under experimental conditions, pig susceptibility to infection with avian influenza A (H5N1) viruses is low (23). Nevertheless, the pandemic (H1N1) 2009 virus is a reassortant that originated from 4 genetically distinct viruses and appeared to be generated in pigs (24), suggesting their role in the generation of pandemic influenza viruses. Infection of pigs with influenza A (H5N1) viruses has been reported in Vietnam (25) and China (26); however, the infection status of pigs in Indonesia remains unknown. We, therefore, explored whether pigs in Indonesia had been infected with influenza A (H5N1) viruses and, if so, whether the viruses were transmitted multiple times and had acquired the ability to recognize human-type receptors.

## Materials and Methods

### Specimen Collection

**Figure 1:**
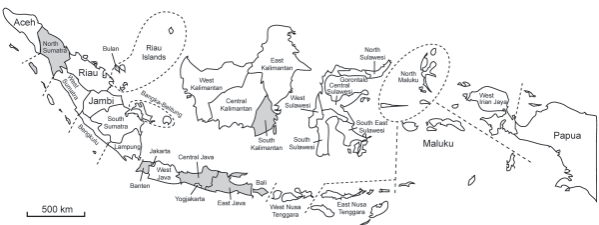
Provinces in Indonesia (gray shading) where surveillance for influenza A (H5N1) virus in pigs was conducted during 2005–2009.

Virologic and serologic surveillance was conducted during 3 rainy seasons during 2005–2009: January–February 2005, October–February 2007, and, November 2008–April 2009. Nasal, fecal, and serum samples were collected from apparently healthy pigs in various districts of Indonesia (Figure 1). The nasal and fecal samples were injected into 10-day-old embryonated eggs, and the allantoic fluid was tested for hemagglutination. Hemagglutination-positive allantoic fluid was subjected to reverse transcription–PCR by using H5 HA–specific and N1 neuraminidase–specific primers; only positive samples were tested further. Serum was analyzed to estimate the prevalence of influenza virus A (H5N1) infection.

### Cells and Virus Isolation

MDCK cells and an MDCK cell line that overexpresses the human β-galactoside α-2,6-sialyltransferase I gene (MDCK-ST6GalI) (27) were maintained in minimal essential medium (MEM) containing 5% newborn calf serum at 37°C in 5% CO_2_. Virus isolation from specimens was performed by using 10-day-old embryonated chicken eggs, MDCK cells, or MDCK-ST6GalI cells in MEM containing 0.3% bovine serum albumin (BSA) (Sigma-Aldrich, Inc., St. Louis, MO, USA) (Table A1). Viruses isolated in MDCK cells were used whenever they were available.

### Serologic Analysis

Swine serum samples were tested for neutralizing antibodies against influenza A/swine/East Java/UT6040/2007 (H5N1) and A/duck/Czechoslovakia/56 (H4N6) viruses. Subtype H4N6 was used as a negative control. Serum was mixed with 3 volumes of receptor-destroying enzyme (Denka Seiken Co., Ltd, Tokyo, Japan) overnight at 37°C and inactivated at 56°C for 30 min. A 2-fold serial dilution series of serum (1:4–1:512) was mixed with an equal volume of influenza virus at 100 TCID_50_ (50% tissue culture infectious doses) and incubated at 37°C for 30 min. Viruses were inoculated to monolayers of MDCK cells for 1 h, washed 2×, and incubated with MEM containing 0.3% BSA for 2 d at 37°C in a 5% CO_2_ incubator. Cytopathic effects were observed to determine the neutralizing activity of the test serum. The detection limit for the neutralizing antibody was <4 dilutions of serum.

### Sequence Analysis

To characterize the swine influenza A (H5N1) viruses isolated in Indonesia, we sequenced the HA genes of 39 viruses isolated from pigs in Banten, East Java, North Sumatra, and South Kalimantan provinces and grouped them according to their genetic similarities. Viral RNA was extracted with ISOGEN (Nippon Gene, Tokyo, Japan) according to the manufacturer’s instructions. Extracted RNA was reverse transcribed with SuperScript III reverse transcriptase (Invitrogen, Carlsbad, CA, USA) and an oligonucleotide complementary to the 12-nt sequence at the 3′ end of the viral RNA and amplified by PCR with Pfu-ultra (Stratagene, La Jolla, CA, USA) or Phusion (Finnzymes, Espoo, Finland) high-fidelity DNA polymerase and primers specific for each segment of the influenza virus A (H5N1). Primer sequences are available upon request. The PCR products were separated by agarose gel electrophoresis, purified by using a MinElute Gel Extraction Kit (QIAGEN, Hilden, Germany), and then sequenced. The nucleotide sequences obtained in this study are available from GenBank, accession nos. HM440051–HM440154.

### Phylogenetic Analysis

We phylogenetically analyzed 13 representative swine influenza A (H5N1) viruses for all 8 viral genes and compared these sequences with publicly available sequences. All sequences were assembled and edited with BioEdit 7 software (28). Neighbor-joining tree analysis was conducted by using ClustalW (www.clustal.org). Estimates of the phylogenies were calculated by performing 100 neighbor-joining bootstrap replicates.

### Receptor Specificity Assays

During replication in pigs, avian influenza viruses may adapt to recognize human-type receptors because such receptors are present in the epithelial cells of pig trachea (18). We therefore analyzed the receptor specificity of representative viruses from each of the 3 swine groups: A/swine/Banten/UT3081/2005 for the 2005 swine group, A/swine/East Java/UT6012/2007 for the 2006–07 swine (A) group, and A/swine/Banten/UT6001/2006 for the 2006–07 swine (B) group. We also analyzed A/swine/Banten/UT3062/2005 clone 6 and A/swine/Banten/3063/05 clone 1, each of which possesses a single amino acid change in HA that distinguishes it from other clones. The receptor specificity of these influenza A (H5N1) viruses was determined by use of an assay that measures direct binding to sialylglycopolymers possessing either SAα2,3Gal or SAα2,6Gal. We used this solid-phase binding assay with the sodium salts of sialylglycopolymers (poly α-l-glutamic acid backbones containing *N*-acetylneuraminic acid linked to galactose through either an α-2,3 or -2,6 bond (Neu5Acα2,3Galβ1,4GlcNAcβ-pAP and Neu5Aαc2,6Galβ1,4GlcNAcβ-pAP) as described (29,30). Briefly, microtiter plates (Polystyrene Universal-BIND Microplate, Corning, NY, USA, USA) were incubated with glycopolymer in phosphate buffered saline (PBS) at 4°C for 3 h and then irradiated under UV light at 254 nm for 2 min. After removal of the glycopolymer solution, the plates were blocked with 0.1 mL PBS containing 2% BSA (Invitrogen) at room temperature for 1 h. After being washed 5× with PBS, the plates were incubated in a solution containing influenza virus (128 hemagglutination units in PBS) at 4°C for 12 h. After 3 more washes with PBS, antibody to the virus was added to the plates, which were then incubated for 2 h at 4°C, washed 3× with ice cold PBS, and then incubated with horseradish peroxidase–conjugated protein A (Organon Teknika N.V; Cappel Products, Turnhout, Belgium; 2000-fold dilution in PBS) at 4°C. After being washed 4× with ice-cold PBS, the plates were then incubated with *o*-phenylenediamine (Sigma-Aldrich) in PBS containing 0.01% H_2_O_2_ for 10 min at room temperature, and the reaction was stopped by adding 0.05 mL of 1N HCl. Absorbance was determined at 490 nm.

## Results

### Virus Prevalence

Of 702 nasal swabs, 52 (7.4%) collected in 2005–2007 yielded influenza A (H5N1) viruses (Table 1; Table A1); no virus was isolated from fecal samples of the same pigs. All 35 viruses isolated in 2005 were from 5 pig farms in the Tangerang District of Banten Province, near an area in which an influenza A (H5N1) outbreak among poultry had been confirmed in 2004 (31) and where the virus has since remained enzootic. Samples collected from a slaughterhouse in the Surabaya district of East Java Province were negative for influenza virus A (H5N1). In the subsequent surveillance period, October 2006–February 2007, we detected viruses in pigs on 4 farms in the Tangerang, Kediri, and Medan districts of Banten, East Java, and North Sumatra, respectively, and in slaughterhouses in the Surabaya and Banjarmasin districts of East Java and South Kalimantan; all sites were near previous outbreaks of influenza virus A (H5N1) infection among poultry. Pigs from which these viruses were isolated did not show any signs of influenza-like illness at the time of sample collection. During the November 2008–April 2009 surveillance period, virus was not isolated from any nasal swabs from 300 pigs tested. However, the 300 serum samples tested indicated that 3 (1%) pigs had neutralizing antibodies against a subtype H5N1 virus but not subtype H4N6, suggesting limited exposure to influenza A (H5N1) viruses. These positive samples were obtained from a farm in the Malang District of East Java Province; neutralizing titers were 4–16 (Table 1).

**Table 1:** Sites and prevalence of influenza A (H5N1) viruses isolated from pigs, Indonesia*

Surveillance period and location	No. samples/no. pigs†	Viruses isolated, no. (rate)	Management type	Distance from poultry
2005 Jan–2005 Feb				
Banten, Tangerang				
Farm A	41/500	12 (29)	Commercial	On site
Farm B	22/500	6 (27)	Commercial	On site
Farm C	13/50	0	Private	On site
Farm D	18/250	11 (61)	Commercial	On site
Farm E	13/50	0	Private	On site
Farm F	29/250	4 (14)	Commercial	On site
Farm G	23/100	2 (9)	Private	On site
East Java, Surabaya, SH	8/100	0	Private	2 km
Total	167/1,800	35 (21)	NA	NA
2006 Oct–2007 Feb				
Banten, Tangerang				
Farm H	34/150	4 (12)	Commercial	On site
Farm I	15/50	1 (7)	Private	On site
East Java				
Surabaya, SH	95/600–700	8 (8)	Government	1 km
Kediri, farm	30/150	1 (3)	Private	1 km
North Sumatra, Medan, farm	38/400	2 (5)	Commercial	0.5 km
South Kalimantan, Banjarmasin, SH	23/50	1 (4)	Private	On site
Total	235/1,400–1,500	17 (7)	NA	NA
2008 Nov–2009 Apr				
East Java				
Tulungagung				
Farm A	25/900	0	Commercial	1 km
Farm B	29/700	0	Commercial	2 km
Surabaya, SH	40/600–700	0	Government	1 km
Jember, farm	18/400	0	Private	2 km
Malang, farm	39/500	0‡	Private	1 km
Central Java, Solo, farm	15/100	0	Private	5 km
Bali				
Denpasar, SH	99/400	0	Government	2 km
Tabanan, farm	9/300	0	Commercial	0.2 km
Riau Islands, Bulan, farm	26/20,000	0	Commercial	No poultry farms on island
Total	300/23,900–24,000	0	NA	NA
Total	702/27,100–27,300	52 (7.4)	NA	NA

*SH, slaughterhouse; NA, not applicable. †Numbers of pigs on farms are estimates. ‡Three samples were positive for A/swine/East Java/UT6040/2007 (H5N1); neutralizing titers were 4 for 1 sample and 16 for 2 samples.

### Virus Sequences

Among the 39 viruses sequenced, the first group comprised 24 isolates collected in Banten Province during January 2005–February 2005; the HA genes of these viruses were either identical or differed by no more than 2 nt. The second group comprised 9 isolates collected during October 2006–February 2007 and also differed by only 2 nt, although the viruses were collected in different provinces: Banten, East Java, North Sumatra, and South Kalimantan. The HA genes in these 2 groups differed from each other by 49–53 nt. The third group included 6 isolates collected in Banten, East Java, and North Sumatra during October 2006–February 2007; these HAs were identical except for 1 nt and differed from those of the first and second groups by 42–45 and 58–61 nt, respectively. Thus, the swine influenza A (H5N1) viruses collected in our surveillance study could be classified into 3 distinct groups on the basis of their HA gene sequences, irrespective of the province from which they were isolated, suggesting extensive movement of pigs among provinces.

### Phylogeny

**Figure 2:**
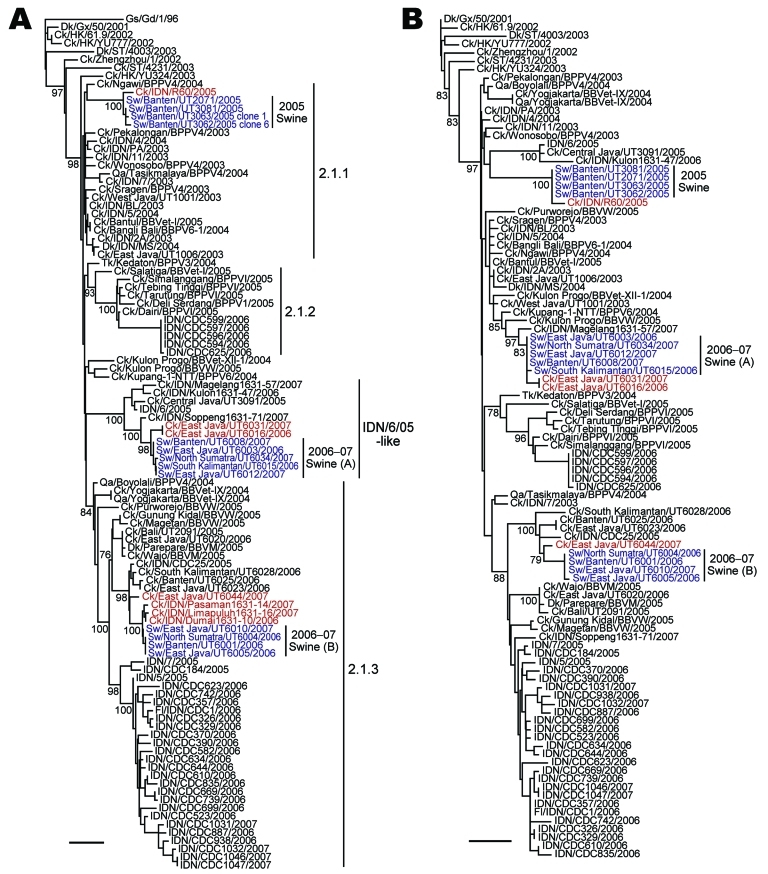
Phylogenetic relationships among the A) hemagglutinin (HA) and B) neuraminidase (NA) genes of influenza A (H5N1) viruses isolated in Indonesia. The numbers below or above the branch nodes indicate neighbor-joining bootstrap values. Analysis was based on nucleotides 281–1675 of the HA gene and 43–1037 of the NA gene. The HA and NA gene trees were rooted to A/goose/Guangdong/1/96 and A/duck/Guangxi/50/2001, respectively. Colors indicate swine viruses (blue) and chicken viruses (red) most closely related to swine viruses. Scale bars indicate 0.01 nt substitutions per site. Ck, chicken; Dk, duck; Fl, feline; Gd, Guangdong; Gs, goose; Gx, Guangxi; HK, Hong Kong; IDN, Indonesia; ST, Shantou; Sw, swine; Tk, turkey; Qa, quail.

**Figure A1:**
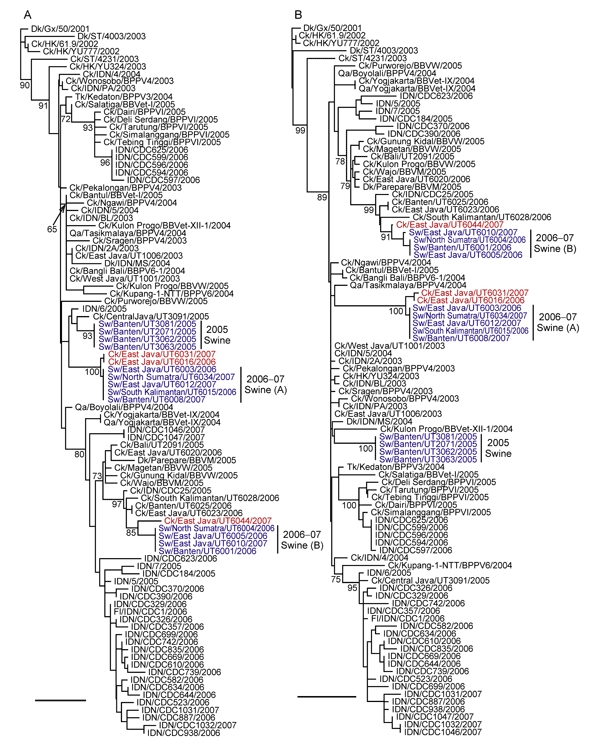
Phylogenetic relationships of the polymerase basic protein (PB) 2 (A) and PB1 (B) genes of influenza A (H5N1) viruses in Indonesia. All trees were generated by neighbor-joining in ClustalW (www.clustal.org). Numbers above or below branches indicate neighbor-joining bootstrap values. Analyses were based on nucleotides 1062–1923 (862 bp) and 94–1485 (1392 bp) of the PB2 and PB1 genes, respectively. Each tree was rooted to A/duck/Guangxi/50/2001. Colors indicate swine isolates (blue) and chicken isolates (red) most closely related to swine viruses. Scale bar indicates 0.01 nt substitutions per site. Ck, chicken; Dk, duck; Fl, feline; Gx, Guangxi; HK, Hong Kong; IDN, Indonesia; Qa, quail; ST, Shantou; Sw, swine.

**Figure A2:**
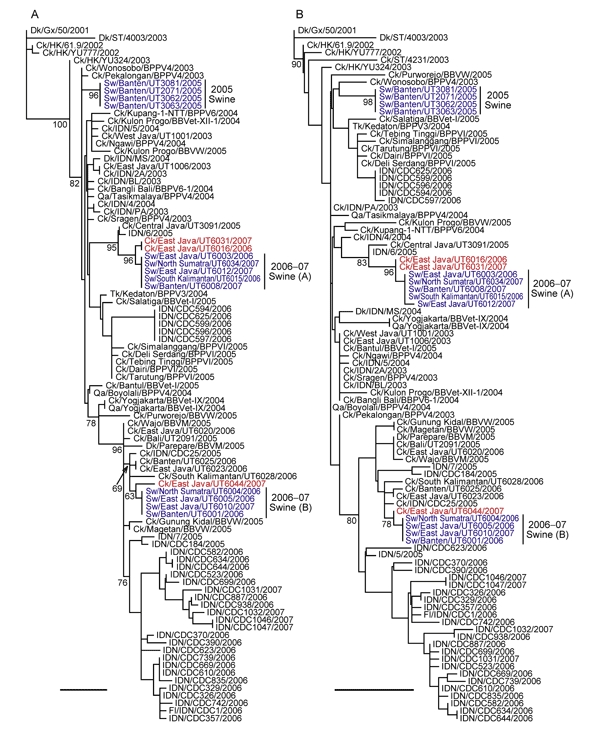
Phylogenetic relationships of the polymerase acidic protein (PA) (A) and nucleocapsid protein (NP) (B) genes of influenza A (H5N1) viruses in Indonesia. All trees were generated by neighbor-joining in ClustalW (www.clustal.org). Numbers above or below branches indicate neighbor-joining bootstrap values. Analyses were based on nucleotides 1426–2172 (747 bp) and 46–913 (868 bp) of the PA and NP genes, respectively. Each tree was rooted to A/duck/Guangxi/50/2001. Colors indicate swine isolates (blue) and chicken isolates (red) most closely related to swine viruses. Scale bar indicates 0.01 nt substitutions per site. Ck, chicken; Dk, duck; Fl, feline; Gx, Guangxi; HK, Hong Kong; IDN, Indonesia; Qa, quail; ST, Shantou; Sw, swine.

**Figure A3:**
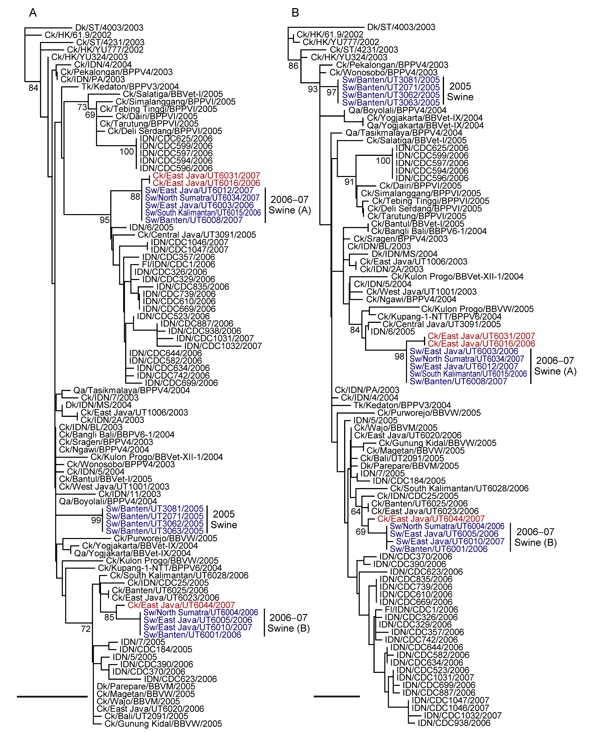
Phylogenetic relationships of the M (A) and NS (B) genes of H5N1 influenza viruses in Indonesia. All trees were generated by the neighbor-joining method in ClustalW (www.clustal.org). Numbers above or below branches indicate neighbor-joining bootstrap values. Analyses were based on nucleotides 77-955 (879 bp) and 64-789 (726 bp) of the M and NS genes, respectively. Each tree was rooted to A/duck/Shantou/4003/03 for M and NS. Colors indicate swine isolates (blue) and chicken isolates (red) most closely related to swine viruses. Scale bar indicates 0.01 nt substitutions per site. Ck, chicken; Dk, duck; Fl, feline; Gx, Guangxi; HK, Hong Kong; IDN, Indonesia; Qa, quail; ST, Shantou; Sw, swine.

Phylogenetic analysis of the HA genes of the 13 representative viruses identified the same 3 groups described above. The HA genes of 4 viruses isolated in 2005 (2005 swine group) were placed in clade 2.1.1, and of the remaining 9 swine viruses isolated during 2006–2007, five were classified into the IDN/6/05-like sublineage (2006–07 swine [A] group) and 4 into clade 2.1.3 (2006–07 swine [B] group) (Figure 2, panel A). The most closely related strains of each swine virus group were chicken influenza A (H5N1) viruses: A/chicken/Indonesia/R60/2005 for the 2005 swine group, A/chicken/East Java/UT6016/2006 and A/chicken/East Java/UT6031/2007 for the 2006–07 swine (A) group, and A/chicken/East Java/UT6044/2007 for the 2006–07 swine (B) group. Analyses of the other 7 genes demonstrated that the phylogenetic relationships established for the HA gene were maintained; that is, the swine viruses in each group possessed nearly identical genes, and each group of swine viruses was most closely related to a chicken virus isolated near the site where the swine viruses were collected (Figure 2, panel B; Figure A1, Figure A2, Figure A3). Our results suggest that influenza A (H5N1) viruses were transmitted from avian species to pigs on at least 3 occasions.

### Receptor Specificity

**Figure 3:**
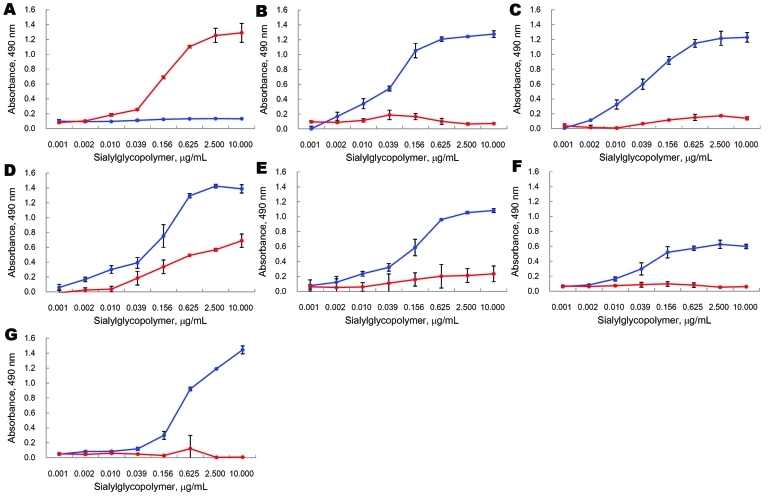
Receptor-binding activity of influenza A (H5N1) viruses. Direct binding of viruses to sialylglycopolymers containing either α2,3-linked (blue) or α2,6-linked (red) sialic acids was measured. A) Human isolate A/Kawasaki/173/2001; B) avian isolate A/chicken/Central Java/UT3091/2005; C) swine isolate A/swine/Banten/UT3081/2005; D) swine isolate A/swine/Banten/UT3062/2005 clone 6; E) swine isolate A/swine/Banten/UT3063/2005 clone 1; F) swine isolate A/swine/East Java/UT6012/2007; G) swine isolate A/swine/Banten/UT6001/2006. Results represent means ± SDs of triplicate experiments.

Sequence analysis of the PCR products of the HA genes of A/swine/Banten/UT3062/2005 and A/swine/Banten/UT3063/2005 indicated that nucleotides were heterogeneous at certain positions, prompting us to plaque purify the viruses in MDCK cells to obtain viral clones with distinct HA sequences (Table 2). We found that most of the swine influenza subtype H5N1 isolates bound to only SAα2,3Gal, whereas the plaque-purified clone 6 of A/swine/Banten/UT3062/05 bound to SAα2,3Gal and SAα2,6Gal (Figure 3), indicating that during their replication in pigs, avian influenza A (H5N1) viruses can acquire the ability to recognize human virus receptors.

**Table 2:** Phylogeny of influenza A (H5N1) viruses isolated from pigs, Indonesia, 2005–2009

Hemagglutinin clade and virus	Group*	Chicken isolates with related genes
Clade 2.1.1		
A/swine/Banten/UT2071/2005	2005 swine	A/chicken/Indonesia/R60/2005†
A/swine/Banten/UT3062/2005‡		
A/swine/Banten/UT3063/2005§		
A/swine/Banten/UT3081/2005		
IDN/6/05-like clade		
A/swine/East Java/UT6003/2006	2006–07 swine (A)	A/chicken/East Java/UT6016/2006 and A/chicken/East Java/UT6031/2007
A/swine/South Kalimantan/UT6015/2006		
A/swine/North Sumatra/UT6034/2007		
A/swine/Banten/UT6008/2007		
A/swine/East Java/UT6012/2007		
Clade 2.1.3		
A/swine/Banten/UT6001/2006	2006–07 swine (B)	A/chicken/East Java/UT6044/2007
A/swine/North Sumatra/UT6004/2006		
A/swine/East Java/UT6005/2006		
A/swine/East Java/UT6010/2007		

*See Figure 2, panel A. †Only hemagglutinin and neuraminidase genes are available through the public database. ‡Of 5 plaque-purified clones, 3 possessed serine at position 134 of the hemagglutinin molecule according to H5 numbering. §Of 5 plaque-purified clones, 2 possessed tryptophan at position 145 of the hemagglutinin molecule according to H5 numbering.

## Discussion

In contrast to the few reported cases of infection of pigs with highly pathogenic avian influenza A (H5N1) viruses (17,25,26), our surveillance study of 7 provinces in Indonesia during 3 periods shows that 7.4% of pigs surveyed during 2005–2007, but not 2008–2009, were infected with influenza A (H5N1) viruses. Phylogenetic analysis indicated that the viruses were transmitted to pigs on several different occasions, probably from poultry on nearby farms. According to the most recent classification of the HA gene (32,33), all avian and human influenza A (H5N1) viruses isolated in Indonesia belong to clade 2.1, which includes 3 well-defined lineages (clades 2.1.1–2.1.3) and a developing lineage termed IDN/6/05-like sublineage. In our study, all 24 viruses isolated during the first surveillance period belonged to the same cluster in clade 2.1.1 (2005 swine group) on the basis of recent HA classification (32,33). The 9 viruses collected during the second surveillance period belonged exclusively to the IDN/6/05-like sublineage, and the 6 remaining viruses collected during the same season were classified into clade 2.1.3; 2006–07 swine (A) and 2006–07 swine (B) groups, represented, respectively, by A/swine/East Java/UT6012/2007 and A/swine/Banten/UT6001/2006. Although no virus was isolated during the third surveillance period, 2008–09, a total of 3 (1%) pigs had neutralizing antibodies against influenza virus A (H5N1). These findings show that although influenza A (H5N1) viruses may not have been extensively circulating in pigs in Indonesia recently, these animals are susceptible to influenza A (H5N1) viruses and can serve as asymptomatic reservoirs for these viruses.

Because the phylogenetic relationships established for the HA gene extended to all viral genes, we conclude that the 3 groups of viruses identified in this survey were likely established independently, suggesting at least 3 separate avian-to-pig episodes of transmission of influenza A (H5N1) viruses during 2005–2009 in Indonesia. Our findings confirm sporadic reports of the susceptibility of pigs to influenza A virus (H5N1) infection in natural (25,26) and experimental settings (23,34) and suggest that when an outbreak of influenza A virus (H5N1) infection occurs on poultry farms, pigs on nearby farms should be evaluated for infection.

We also found evidence of pig-to-pig transmission of influenza A virus (H5N1), particularly among animals sampled during the first surveillance period. Many viruses possessing almost identical genes were isolated from pigs on the same farms (Table 1, Table 2). Pig-to-pig transmission would likely prolong the duration of influenza A (H5N1) virus infection within a pig population, thereby increasing the likelihood of adaptation and the subsequent generation of influenza A (H5N1) viruses that replicate efficiently in humans.

The lack of influenza-like signs in pigs infected with influenza A (H5N1) viruses has several public health implications. In Indonesia, pigs are transported to different locations according to market needs. This movement is reflected in our finding that clusters of swine viruses collected after 2006 were not consistent with those common to the sampling region. Indeed, viruses collected in North Sumatra, South Kalimantan, East Java, and Banten provinces showed identical or nearly identical genes, indicative of extensive transport of infected pigs throughout Indonesia. Thus, pathogenic influenza A (H5N1) viruses could easily evade detection as they spread through Indonesia in asymptomatic pigs being transported from province to province.

Our analysis of viral receptor specificities showed that 1 plaque-purified clone of A/swine/Banten/UT3062/2005 bound to avian-type and human-type receptors. Serine at position 134 was responsible for the human-type receptor recognition. This position is located within the 130-loop structural component of the receptor-binding pocket (35). Hence, the amino acid change at this position may affect receptor binding. Because serine at position 134 is never seen in avian influenza A (H5N1) viruses (alanine is highly conserved at this position in avian influenza A [H5N1] viruses), the Ala134Ser mutation probably occurred during adaptation of the virus to pigs. According to a previous report (36), human isolates possessing valine at this position could also bind to the human-type receptor, although a mutation at position 129 (L129V) was also required for the human-type receptor recognition in this strain. Therefore, mutations at position 134 probably correlated with human-type receptor recognition and may serve as molecular markers for assessing the pandemic potential of influenza virus A (H5N1) isolates.

Although influenza virus A (H5N1) infection was not reported among swine workers in Indonesia while we were collecting our pig specimens, a previous cohort study showed that such workers, as well as their unexposed spouses, had increased levels of antibody to swine influenza A (H1N1) viruses (37), suggesting that humans are indeed susceptible to swine-adapted viruses (38). The recent swine-origin pandemic (H1N1) 2009 further demonstrates that pigs can be a potential source of virus capable of causing a human influenza pandemic (24). These findings suggest that as influenza A (H5N1) viruses spread among pigs and adapt to recognize human-type receptors, farmers, swine workers, and their families will be at greatest risk for infection by the newly adapted viruses.

In summary, we found that influenza A (H5N1) viruses have been transmitted multiple times to pig populations in Indonesia and that 1 virus has acquired the ability to recognize human-type receptors. Of particular concern is that pigs infected with influenza A (H5N1) viruses showed no significant influenza-like signs and were likely transported to and from different provinces in Indonesia. On the basis of our findings, we encourage the Indonesian government to control the transport of pigs within Indonesia. Otherwise, opportunities for this avian virus to adapt to mammals will increase, as will the risk for emergence of a new pandemic influenza virus.

## Appendix

**Table A1:** Virus strains from pigs, Indonesia

Surveillance period	Sampling province	Location	Virus	Isolated in
2005 Jan–Feb	Banten	Tangerang Farm A	A/swine/Banten/UT2003/2005	Egg
			A/swine/Banten/UT2004/2005	Egg
			A/swine/Banten/UT2005/2005	Egg
			A/swine/Banten/UT2009/2005	Egg
			A/swine/Banten/UT2010/2005	Egg
			A/swine/Banten/UT2012/2005	Egg
			A/swine/Banten/UT3069/2005	MDCK
			A/swine/Banten/UT3071/2005	MDCK-ST6GalI
			A/swine/Banten/UT3072/2005	MDCK
			A/swine/Banten/UT3073/2005	MDCK-ST6GalI
			A/swine/Banten/UT3075/2005	MDCK
			A/swine/Banten/UT3076/2005	MDCK-ST6GalI
		Tangerang Farm B	A/swine/Banten/UT3061/2005	MDCK
			A/swine/Banten/UT3062/2005	MDCK
			A/swine/Banten/UT3063/2005	MDCK
			A/swine/Banten/UT3064/2005	MDCK
			A/swine/Banten/UT3065/2005	MDCK
			A/swine/Banten/UT3066/2005	MDCK
		Tangerang Farm D	A/swine/Banten/UT2035/2005	Egg
			A/swine/Banten/UT3081/2005	MDCK
			A/swine/Banten/UT3082/2005	MDCK
			A/swine/Banten/UT3083/2005	MDCK
			A/swine/Banten/UT3084/2005	MDCK
			A/swine/Banten/UT3085/2005	MDCK
			A/swine/Banten/UT3086/2005	MDCK
			A/swine/Banten/UT3087/2005	MDCK
			A/swine/Banten/UT3088/2005	MDCK
			A/swine/Banten/UT3089/2005	MDCK
			A/swine/Banten/UT3090/2005	MDCK
		Tangerang Farm F	A/swine/Banten/UT2058/2005	Egg
			A/swine/Banten/UT2069/2005	Egg
			A/swine/Banten/UT2070/2005	Egg
			A/swine/Banten/UT2071/2005	Egg
		Tangerang Farm G	A/swine/Banten/UT2027/2005	Egg
			A/swine/Banten/UT2028/2005	Egg
2006 Oct– 2007 Feb	Banten	Tangerang Farm H	A/swine/Banten/UT6001/2006	Egg
			A/swine/Banten/UT6002/2006	Egg
			A/swine/Banten/UT6008/2007	Egg
			A/swine/Banten/UT6009/2007	Egg
		Tangerang Farm I	A/swine/Banten/UT6007/2007	Egg
	East Java	Surabaya Slaughterhouse	A/swine/East Java/UT6003/2006	Egg
			A/swine/East Java/UT6006/2006	Egg
			A/swine/East Java/UT6010/2007	Egg
			A/swine/East Java/UT6011/2007	Egg
			A/swine/East Java/UT6012/2007	Egg
			A/swine/East Java/UT6040/2007	MDCK
			A/swine/East Java/UT6043/2007	MDCK
			A/swine/East Java/UT6049/2007	MDCK
		Kediri Pig Farm	A/swine/East Java/UT6005/2006	Egg
	South Kalimantan	Banjarmasin Slaughterhouse	A/swine/South Kalimantan/UT6015/2006	Egg
	North Sumatra	Medan Farm	A/swine/North Sumatra/UT6004/2006	Egg
			A/swine/North Sumatra/UT6034/2007	Egg
